# Separation and detection of Gram-negative bacteria via vancomycin-functionalized magnetic beads and aminopeptidase test strips

**DOI:** 10.3389/fbioe.2025.1712799

**Published:** 2025-11-20

**Authors:** Xiao Xu, Anji Zhu, Lixia Bai, Shihong Li, Wenbo Ge, Yajun Yang, Xiwang Liu, Zhe Qin, Zhun Li, Jianyong Li

**Affiliations:** 1 Key Lab of New Animal Drug of Gansu Province, Key Lab of Veterinary Pharmaceutical Development of Ministry of Agriculture and Rural Affairs, Lanzhou Institute of Husbandry and Pharmaceutical Sciences of CAAS, Lanzhou, China; 2 College of Veterinary Medicine, Gansu Agricultural University, Lanzhou, China

**Keywords:** biosensor, vancomycin-functionalized magnetic beads, aminopeptidase test, Gram-negative bacteria, point-of-care-testing, milk contamination

## Abstract

**Introduction:**

Gram-negative bacteria are key pathogens causing food contamination and animal diseases (such as bovine mastitis), and they are prone to developing multidrug resistance, necessitating rapid and sensitive detection technologies.

**Methods:**

We developed a novel detection method based on an antibiotic affinity strategy, which combines vancomycin-functionalized magnetic beads (Van-MBs) with aminopeptidase test strips. Vancomycin, which binds exclusively to D-alanyl-D-alanine (D-Ala-D-Ala) on the cell wall of Gram-positive bacteria, is used by Van-MBs to capture Gram-positive bacteria from the sample. After magnetic separation, the supernatant containing Gram-negative bacteria is retained. This supernatant reacts with aminopeptidase test strips, utilizing the enzyme's unique properties as a Gram-negative enzyme. Visual detection is made possible by the enzyme's catalysis of the chromogenic substrate, which produces a yellow signal.

**Results:**

Using raw milk as a representative sample, validation showed that the approach achieves a detection limit of 1.0 × 10^1^ CFU/mL within 100 minutes.

**Discussion:**

This approach is quick, sensitive, and visual, and it doesn't require complicated equipment. It also offers useful technical support for safe food production, early animal disease detection, and sensible antibiotic administration.

## 1 Introduction

Milk and dairy products serve as fundamental nutritional sources for global populations, yet their biosafety presents persistent challenges in food biotechnology. Despite modern sterilization techniques effectively ensuring the safety of processed dairy products (Wei et al., 2023), raw milk contamination-particularly from pathogens associated with bovine mastitis-remains a critical One Health concern at the animal-human-environment interface (Nelson et al., 2024; Qi et al., 2024). Therefore, the development of rapid, on-farm diagnostic platforms is imperative. Compared to Gram-positive bacteria, Gram-negative bacteria have more complicated and variable resistance mechanisms, and multi-drug resistance issues are more prevalent (Breijyeh et al., 2020). Thus, it is crucial to identify Gram-negative pathogenic bacteria as soon as possible and to administer focused pharmacological therapy.

Gold-standard culture methods require 24–72 h for microbial isolation and phenotypic characterization, rendering them ineffective for on-farm decision-making. Immunoassays offer high sensitivity and specificity compared to these culture methods (Jaeger et al., 2017; Pang et al., 2018). However, it is challenging and expensive to generate the necessary specific antibodies. While nucleic acid-based molecular biology techniques are effective for quick detection (Cornelissen et al., 2016; Sheet et al., 2016), the extraction of bacterial nucleic acids is labor-intensive and equipment-demanding. Further advancements include sensitive electrochemical biosensors based on molecular imprinting (Liu et al., 2025). Despite their sensitivity, the requirement for complex electrode modification and specialized apparatus often limits their practicality for point-of-care settings. Beyond detection, innovative theranostic strategies, such as bactericidal magnetic silica hexapods, exemplify the fusion of diagnosis and therapy (Quan et al., 2024). However, their broad-spectrum mechanism fails to provide the pathogen-specific information that is vital for guiding antibiotic selection. Therefore, developing a detection method that can specifically, visibly, and easily identify Gram-negative bacteria in complex samples is indispensable for achieving precision medication in clinical environments.

In contrast to these complex strategies, an alternative approach uses the inherent specificity of antibiotic-based recognition. Recent advances in antibiotic-functionalized biosensors have demonstrated the potential of molecular recognition engineering for bacterial detection (Wang et al., 2017; 2019; Su et al., 2018). Among the recognition molecules derived from antibiotics, vancomycin belongs to the glycopeptide antibiotics. Vancomycin has the ability to attach firmly to the D-alanyl-D-alanine (D-Ala-D-Ala) dipeptide component of the cell wall of Gram-positive bacteria via five hydrogen bonds, which inhibits the synthesis of the bacterial cell wall and has an antibacterial impact on Gram-positive bacteria (Hubbard and Walsh, 2003; Kell et al., 2008). On the other hand, vancomycin is difficult to pass through the outer membrane of Gram-negative bacteria and limits its ability to bind to D-Ala-D-Ala, which leads to its ineffectiveness against these Gram-negative bacteria (Kell et al., 2008). This characteristic makes vancomycin a useful recognition element for ensnaring Gram-positive bacteria. L-alanine aminopeptidase is an enzyme localized on the bacterial cell membrane. This enzyme is ubiquitous in Gram-negative bacteria but almost absent in Gram-positive bacteria. Therefore, the ability to distinguish between Gram-positive and Gram-negative bacteria can be achieved through the detection of L-alanine aminopeptidase activity (Cellier et al., 2014). L-alanine-4-nitroanilide can break down into 4-nitroaniline and L-alanine in the presence of L-alanine aminopeptidase, and 4-nitroaniline turns yellow (Carlone et al., 1982).

**FIGURE 1:**
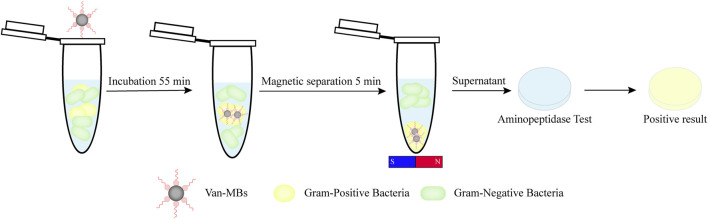
Diagram illustrating a rapid detection method for Gram-negative bacteria using vancomycin-functionalized magnetic beads and the aminopeptidase test.

In this study, we integrated two distinct identification methods mentioned above to establish a quick way to identify Gram-negative bacteria. Briefly, Gram-positive bacteria are identified and concentrated using magnetic beads functionalized with vancomycin (Van-MBs). Gram-negative bacteria are retained in the supernatant, while Gram-positive bacteria are contained in the magnetic complex. Using an aminopeptidase test to detect the supernatant, the test strip will turn yellow if there are Gram-negative bacteria present (Figure 1). As a point-of-care testing (POCT) platform, this method provides a quick, simple, and visually appealing approach for detecting Gram-negative bacteria, thereby aiding in the early diagnosis and rational use of drugs for raw milk.

## 2 Materials and methods

### 2.1 Reagents and bacteria strains

Carboxyl MBs with a diameter of 300 nm were purchased from Beaver Biomedical Engineering Co. Ltd. (Suzhou, Jiangsu, China). 1-(3-dimethylaminopropyl)-3-ethylcarbodiimide hydrochloride (EDC), N-hydroxysuccinimide (NHS), and vancomycin were purchased from Aladdin (Shanghai, China). Fluorescein isothiocyanate (FITC), bovine serum albumin (BSA), Tween-20, dimethyl sulfoxide (DMSO) and 1×phosphate buffered solution (PBS, pH = 7.2–7.4) were obtained from Solarbio Science and Technology Co., Ltd. (Beijing, China). L-Alanine 4-nitroanilide hydrochloride was obtained from JSENB (Hong Kong, China). Mueller Hinton (MH) broth medium, MH agar medium, and Chromogenic Staph. aureus Agar were all obtained from Huankai Microbial Sci. &Tech. CO. Ltd. (Guangzhou, Guangdong, China). The MiniBEST Bacteria Genomic DNA Extraction Kit and the Premix Ex Taq^TM^ (Probe qPCR) were both obtained from Takara Bio Inc. (Otsu, Shiga, Japan). The 16-Plex Bovine Mastitis Pathogen Nucleic Acid Detection Kit (Real-Time PCR Method) was purchased from Zhongke iCare (Zhejiang) Biotechnology Co., Ltd.

The *Staphylococcus aureus* (*S. aureus,* ATCC 29213), *Streptococcus agalactiae* (*S. agalactiae*, ATCC 12386), *Streptococcus dysgalactiae* (*S. dysgalactiae*, ATCC 35666), *Klebsiella pneumoniae* (*K. pneumoniae*, ATCC 700603), and *Proteus mirabilis* (*P. mirabilis*, ATCC 12453) were purchased from the *American Type Culture Collection (ATCC)* (Manassas, Virginia, United States). The *Escherichia coli* (*E. coli*, CMCC 44102) was purchased from the National Center for Medical Culture Collection (*CMCC*) (Beijing, China).

### 2.2 Bacteria culture and counting

All bacterial strains were grown in appropriate mediums under recommended culture conditions. After incubation at 37 °C overnight with continuous shaking, the cultured bacteria were then serially diluted 10-fold with PBS. Each bacterial diluent (100 μL) was evenly distributed on the surface of the MH agar plates. Colonies were counted after incubation for 24 h at 37 °C.

### 2.3 Preparation of FITC-Van

1.0 mL of DMSO was used to dissolve 1.0 mg of FITC, and a 500 μL mixture was added to 3 mL of sodium carbonate buffer (100 mM, pH 9.0) that has 1 mg of vancomycin. The reaction was continued for 12 h at 4 °C in the dark under constant shaking. The reaction solution was then dialyzed by a dialysis bag with a cut-off molecular weight of 1000 Da for 24 h at 4 °C to eliminate unreacted FITC.

### 2.4 Fluorescence of bacterial staining by FITC-Van

500 μL of the 1.0 × 10^8^ CFU/mL *S*. *aureus* and *E. coli* culture solution were taken and incubated with 60 μL of vancomycin marked with fluorescein isothiocyanate (FITC-Van) at room temperature in the dark for 15 min. Then the stained bacteria were washed three times by centrifugation for 5 min at 3500 r/min and resuspended in 200 μL of sterile PBS buffer. Ultimately, 10 μL of stained bacterial solution was dropped onto a microscope slide and covered by a coverslip, and a laser confocal microscope (LSM800, Zeiss, Jena, Germany) was used to view the samples. The fluorescence micrographs were captured with an excitation wavelength of 488 nm and an emission wavelength of 525 nm.

### 2.5 Preparation of Van-MBs

100 μL of carboxyl MBs (MBs) were resuspended in 1 mL of PBS after being washed three times with PBS. Then, EDC 0.32 mg, NHS 0.35 mg, and 3 μL Tween-20 were added. The MBs suspension was left on a shaking mixer at room temperature for 1 h to activate the carboxyl on their surface. After three washes, the activated MBs were resuspended in 1 mL of PBS. Following the addition of 25 mg BSA and 4 μL of Tween-20 to MBs, the mixture was shaken continuously for 2 h at room temperature. The mixture was then washed three times to eliminate unbound BSA and resuspended in 1 mL of PBS. Subsequently, 100 mg of vancomycin was dissolved in 1 mL of PBS, and then 169.3 mg of EDC and 47.98 mg of NHS were added to the solution. The vancomycin solution was left on a shaking mixer at room temperature for 10 min. After adding the activated vancomycin to the MBs suspension, the coupling was done for 6 h at room temperature using a shaking mixer. Following the completion of the coupling, the uncoupled vancomycin was removed by washing three times, and the mixture was resuspended in 1 mL of PBS.

### 2.6 Characterization

#### 2.6.1 Characterization of Van-MBs

The hydrated particle size (Mastersizer 2000, Malvern, UK) and zeta potential (Zetasizer Nano ZS90, Malvern, United Kingdom) of both MBs and Van-MBs were measured in order to confirm whether vancomycin was successfully coupled to the surface of MBs. Vancomycin coupled on the surface of Van-MBs was quantitatively analyzed using a UV-visible spectrophotometer (Cary 100 UV-Vis, Agilent, Santa Clara, CA, United States).

#### 2.6.2 Assessment of antibacterial ability

To ascertain whether the Van-MBs can still resist Gram-positive bacteria, 200 µL of each solution containing 1.0 × 10^8^ CFU/mL of *E. coli* and *S. aureus* was plated on the MH plate, respectively. Coat filter paper with 1 mg/mL vancomycin, 10 mg/mL MB, 1 mg/mL Van-MBs, and a blank conduct a plate drug susceptibility test. The results can be observed for the inhibitory zone’s size 24 h after these plates were incubated at 37 °C.

#### 2.6.3 Evaluation of the capacity to bind the bacteria

100 μL of 1.0 × 10^5^ CFU/mL *E. coli* and *S. aureus* were taken and incubated with 100 μL Van-MBs at 37 °C for 55 min. Subsequently, the magnetic complexes were washed three times and resuspended in 100 µL of PBS. Finally, it was placed under a scanning electron microscope (SEM) (JSM-IT700HR, Tokyo, Japan) to observe the binding of Van-MBs to bacteria.

### 2.7 Optimization of detection method for Gram-negative bacteria

#### 2.7.1 Optimization of Van-MBs separation conditions

100 µL of *S. aureus* at a concentration of 1.0 × 10^5^ CFU/mL was incubated for various volumes of Van-MBs (10, 30, 50, 70, 100, 120, and 150 µL) at 37 °C and 200 r/min in an incubator for varying times (15, 25, 35, 45, 55, 65, and 75 min). Then magnetic separation took varying times (3, 4, 5, 6, and 7 min). Both the magnetic complexes and the supernatant were gathered. 100 μL of PBS was used to resuspend the magnetic complexes. The supernatant and resuspended magnetic complexes were evenly spread on the Chromogenic Staph. aureus Agar, and the capture rate was calculated based on the number of colonies on the plate. Capture rate = (number of bacteria in precipitate/number of bacteria in supernatant + number of bacteria in precipitate) × 100%.

#### 2.7.2 Optimization of aminopeptidase test strips

L-alanine-4-nitroaniline hydrochloride was dissolved with PBS at concentrations of 2, 4, 6, 8, 10, and 15 mg/mL. Aspirated different volumes (20, 40, 60, 80, 100, and 150 μL) of varying concentration solutions onto sterile filter strips and subsequently enabled them to dry. For 30 min at 37 °C, incubate 200 μL of 1.0 × 10^8^ CFU/mL *E. coli* on aminopeptidase test strips with varying concentrations. The ideal concentration can be found by the color change of the test strip.

### 2.8 Establishment of detection method for Gram-negative bacteria

#### 2.8.1 Specificity of the detection method

100 µL of *E*. *coli*, *K. pneumoniae*, *P. mirabilis*, *S. aureus*, *S. agalactiae*, and *S. dysgalactiae* at a concentration of 1.0 × 10^5^ CFU/mL was incubated with 100 µL of Van-MBs at 37 °C and 200 r/min in an incubator for 55 min, followed by magnetic separation for 5 min. Both the magnetic complexes and the supernatant were gathered. PBS was used to resuspend the magnetic complexes. Using the MiniBEST Bacteria Genomic DNA Extraction Kit, the supernatant and resuspended magnetic complexes were utilized to extract DNA, which was subsequently amplified using real-time quantitative PCR (qPCR) established in our laboratory. Table 1 displays the primers and probes used in qPCR.

**TABLE 1:** Primers and probes for qPCR.

System	Bacteria	Target gene	Primers and probes	Sequencing (5′-3′)
Gram-positve system	*S. aureus*	*nuc*	Nuc-F	CAGTGCAACTTCAACTAA
			Nuc-R	GTC​TGA​ATG​TCA​TTG​TTT​G
			Nuc-P	FAM-TTAACCGTATCACCATCAATCGCTT- BHQ1
	*S. agalactiae*	*ef-tu*	Eftu-F	ACTGGTGTTGAAATGTTC
			Eftu-R	CACGTTCGATTTCATCAC
			Eftu-P	VIC-AACACCACGAAGAAGAACACCAAC-BHQ1
	*S. dysgalactiae*	*tpch*	Tpch-F	TGC​TCT​TCA​TAA​CAA​TAA​TAA​AC
			Tpch-R	CTG​GAG​GCG​TAA​TAT​TAA​C
			Tpch-P	CY5-TCGTTCTAACCAGTGACCGCA-BHQ2
Gram-negative system	*E. coli*	*phoa*	Phoa-F	GGCTTTCATGGTGTAGAA
			Phoa-R	CAG​TGA​TGG​TGA​TGA​GTT​A
			Phoa-P	FAM-CCGAAGAGGATTCACAAGAACATACC-BHQ1
	*K. pneumoniae*	*rcsa*	Rcsa-F	GTG​CAT​GAT​GAT​GAA​AGT​AA
			Rcsa-R	CGGTCAAAATGGATGTTC
			Rcsa-P	VIC-CATTATTCGCCAGATCATTACGCAA-BHQ1
	*P. mirabilis*	*urer*	Urer-F	CCT​GAG​GTG​GTT​AAA​ACT​A
			Urer-R	GACCCCTTCGTGATAAAC
			Urer-P	CY5-CGTTACTTCAGCAATGTCTACCGC-BHQ2

The resuspended magnetic complexes were evenly spread on the MH for 37 °C and cultured overnight, and the capture rate was calculated based on the number of colonies on the plate.

The supernatant was incubated with 100 μL of 10 mg/mL aminopeptidase test strips at 37 °C for 30 min, and then the color change of the filter strip was observed.

#### 2.8.2 Sensitivity of the detection method

Took 100 μL of 1.0 × 10^7^ to 1.0 × 10^1^ CFU/mL *E. coli*, incubated them for 55 min at 37 °C at 200 r/min with 100 μL of Van-MBs, and then magnetically separated them for 5 min. To evaluate the sensitivity of the method, we incubated the supernatant with 100 μL of 10 mg/mL aminopeptidase test strips for 30 min at 37 °C. Then, watch the color change of the filter strip. For the semi-quantitative colorimetric analysis, photographs of the test strips were captured using a camera (Nikon Z 30, Japan) under consistent white light illumination without flash. The camera was operated in auto mode. These images were saved in RGB format for subsequent analysis. To evaluate the colorimetric response and determine the Limit of Detection (LOD), a semi-quantitative analysis of all test strip images was performed using ImageJ software. Briefly, each RGB image was split into its red, green, and blue channels using ImageJ’s Split Channels function. The grayscale image of the blue channel was selected for analysis. This is because the yellow color of the produce, 4-nitroaniline, is complementary to blue. As the yellow color intensifies, it absorbs more blue light, resulting in a low signal in the blue channel, which provides the highest sensitivity and optimal signal-to-noise ratio for quantification. A fixed-sized rectangle was placed on the reaction zone of each test strip, and the mean gray value was measured. The gray value is defined on an 8-bit scale from 0 (black) to 255 (white). The threshold was determined to follow the 3-sigma criterion. The mean (μ) and standard deviation (σ) of the gray values from the blank controls (n = 3) were calculated. The statistical threshold was then defined by the formula: threshold = μ - 3 × σ. The LOD was defined as the lowest bacterial concentration for which the measured mean gray value was statistically less than the threshold (Gray_Value_sample < threshold).

### 2.9 Clinical sample detection

A total of 63 bovine mastitis milk samples collected from a dairy farm in Gansu Province, China, were subjected to clinical detection. 100 μL of milk samples and 100 μL of Van-MBs were pipetted and mixed with a vortex, then incubated for 55 min at 37 °C and 200 r/min in a shaker. The solution was magnetically separated for 5 min. After incubating the supernatant with the aminopeptidase test strips for 30 min at 37 °C, the test strip’s color change was observed. Following the colorimetric detection, a semi-quantitative grayscale analysis was performed on the test strips from clinical samples using ImageJ software to provide an objective criterion for the visual results. Meanwhile, DNA was extracted from these 63 samples using the MiniBEST Bacteria Genomic DNA Extraction Kit, and the 16-Plex Bovine Mastitis Pathogen Nucleic Acid Detection Kit was used for detection according to the manufacturer’s instructions.

## 3 Results

### 3.1 Characterization of binding capability of vancomycin towards bacteria

**FIGURE 2:**
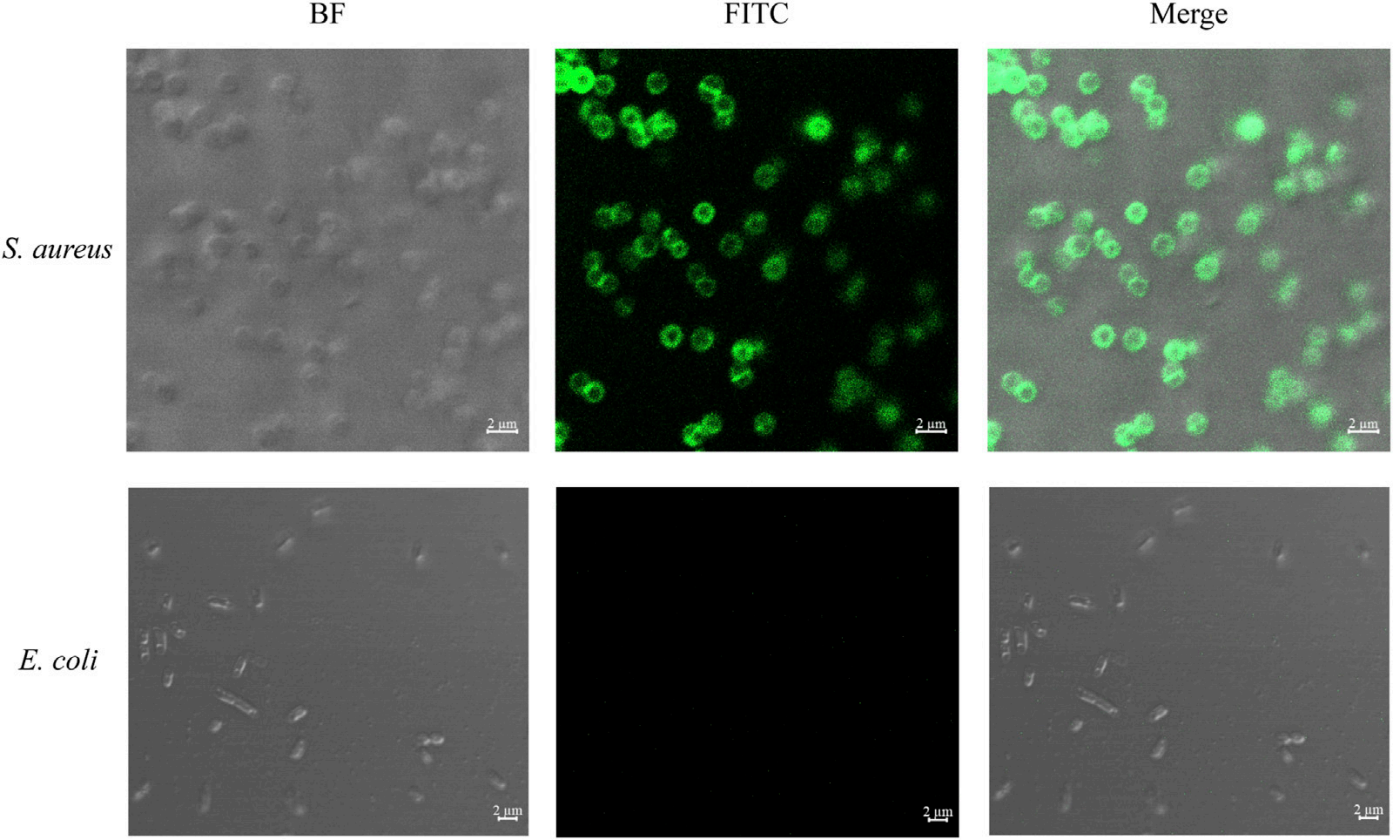
Confocal laser micrographs of bacteria stained with FITC-labeled vancomycin. The specific binding of vancomycin to Gram-positive bacteria validates its use as an effective recognition element for capture.

To determine the viability of utilizing vancomycin as an identification element for Gram-positive bacteria, *S*. *aureus* and *E*. *coli* were stained with FITC-conjugated vancomycin. As shown in Figure 2, when FITC-labeled vancomycin was co-incubated with *S*. *aureus* and *E*. *coli*, the surface of *S*. *aureus* showed strong green fluorescence, whereas *E*. *coli* did not emit light. This result confirmed the significant binding capacity of vancomycin to Gram-positive bacteria.

### 3.2 Characterization of Van-MBs

**FIGURE 3:**
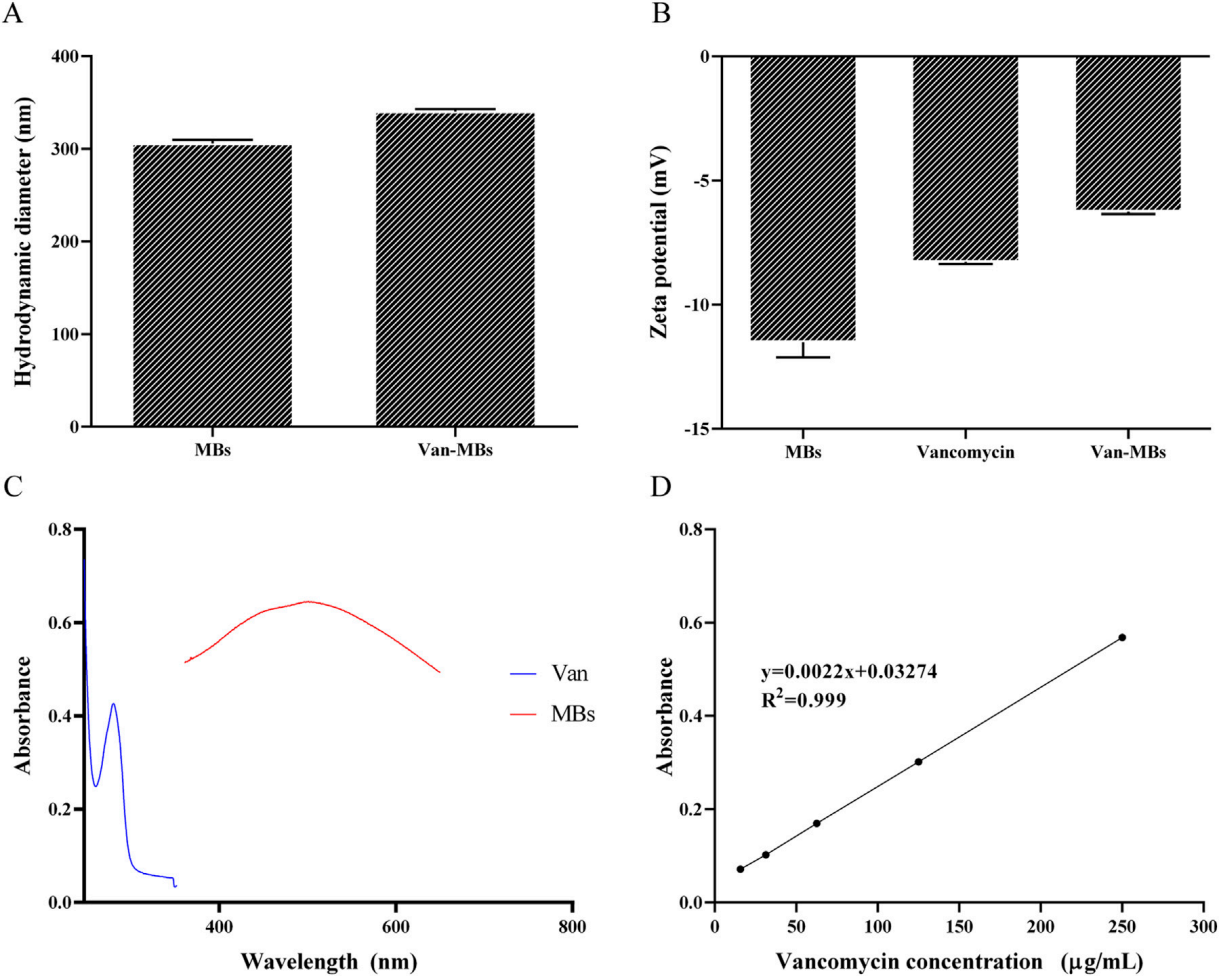
Van-MBs characterization results. **(A)** Hydration particle size, **(B)** Zeta potential, **(C)** Absorption wavelength, **(D)** Standard curve between Van concentration and absorbance.

The zeta potential and hydrated particle size of MBs and Van-MBs were measured in order to confirm that vancomycin is linked to the surface of MBs. As shown in Figure 3A, MBs have a hydrated particle size of 310 nm, and the Van-MBs particle size increases to 342 nm. The zeta potentials of MBs and vancomycin are −11.9 mV and −8.37 mV, respectively, while the zeta potential of Van-MBs rises to −6.34 mV, as seen in Figure 3B. Both the hydrated particle size and zeta potential results indicate that vancomycin has been successfully coupled to the surface of MBs. Moreover, a UV-visible spectrophotometer was used to quantitatively measure the vancomycin coupled to the surface of Van-MBs. Vancomycin’s maximum absorption wavelength is 280 nm, while MBs' maximum absorption wavelength is 501 nm, as illustrated in Figure 3C. As a result, MBs have no effect on vancomycin’s quantification. As can be seen in Figure 3D, the standard curve of vancomycin concentration and absorbance is y = 0.0022x + 0.03274, R^2^ = 0.999. The produced Van-MBs have an absorbance of 0.46 a.u. As the standard equation, the surface vancomycin concentration was 197.20 μg/mL in Van-MBs.

### 3.3 Functional analysis of Van-MBs

**FIGURE 4:**
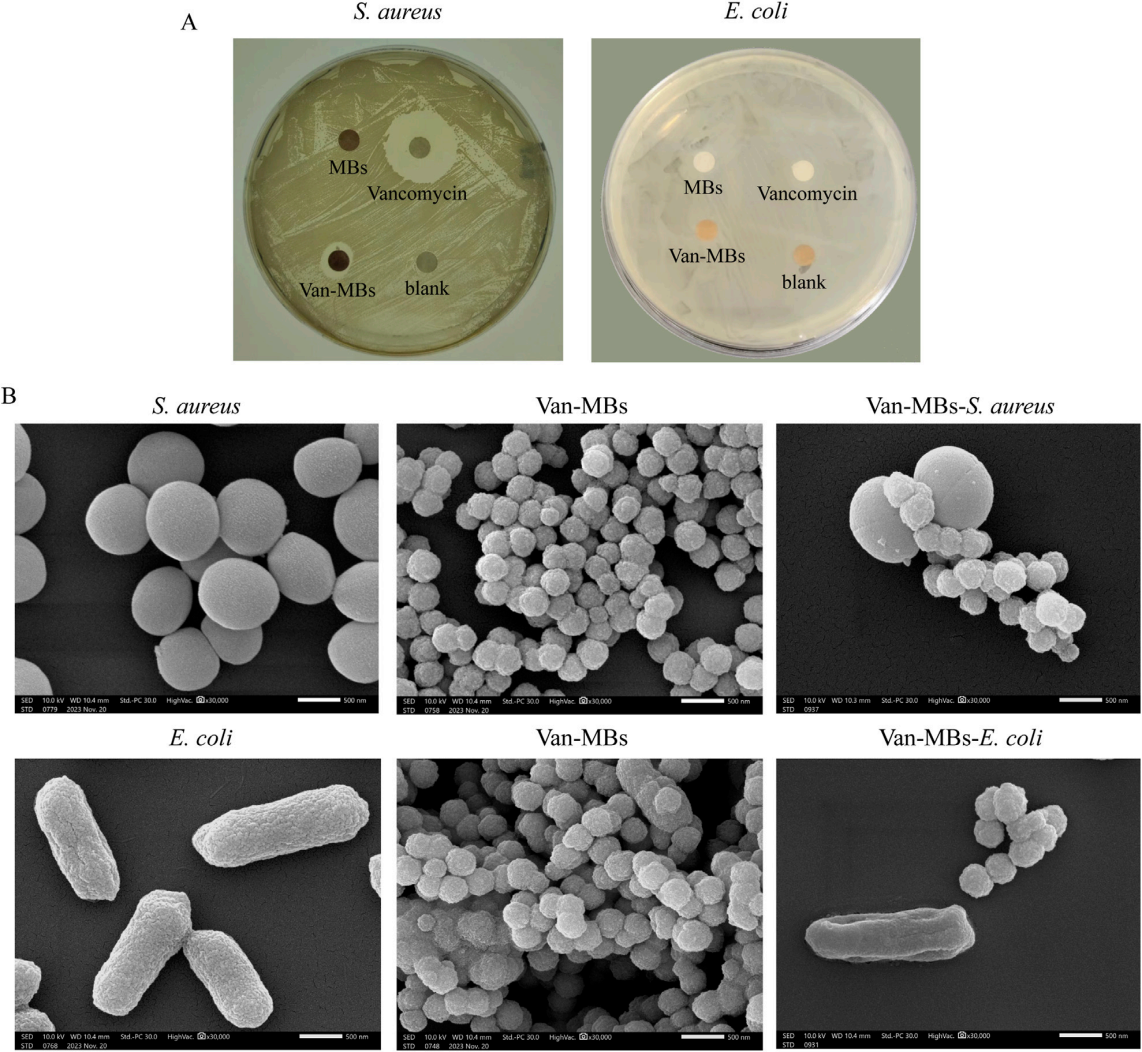
Assessment of the antibacterial ability of Van-MBs. **(A)** Bactericidal halo test of Van-MBs, **(B)** SEM result of Van-MBs binding with bacteria.

MH plates were utilized for the bactericidal halo test to evaluate the antibacterial properties of Van-MBs. As shown in Figure 4A, only inhibition zones developed around the filter strip coated with vancomycin and Van-MBs in *S. aureus*. This suggested that Van-MBs had no effect on *E. coli* but still had an inhibitory effect on *S. aureus*. The binding of Van-MBs to *E. coli* and *S. aureus* was further investigated through SEM. Both *E. coli* and *S. aureus* retained their original cell shapes, and Van-MBs uniformly bonded to *S. aureus*, as demonstrated in Figure 4B. These results demonstrated that Van-MBs can capture Gram-positive bacteria.

### 3.4 Optimization of detection method

**FIGURE 5:**
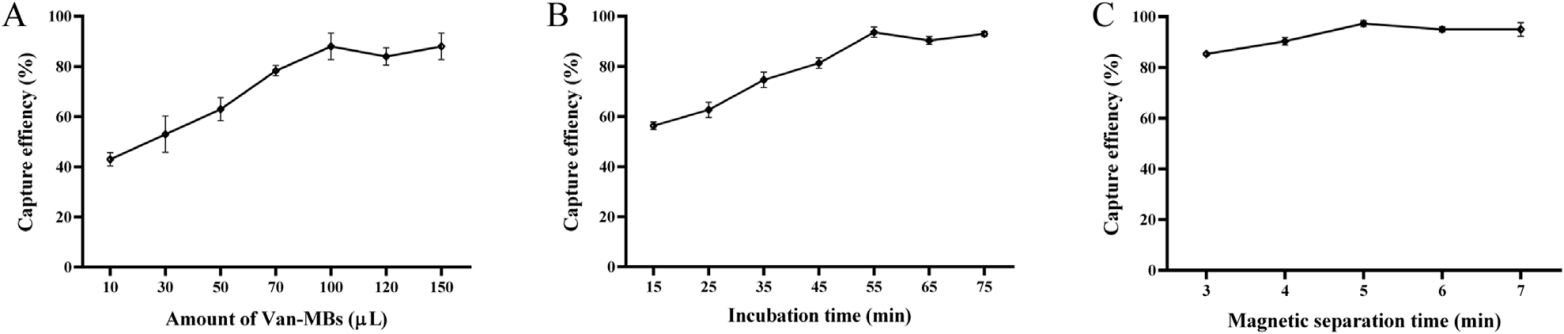
Optimization results of conditions between Van-MBs and bacteria. **(A)** Amount of Van-MBs, **(B)** Incubation time, **(C)** Magnetic separation time. The optimal conditions (100 μL, 55 min, and 5 min, respectively) were determined based on the highest capture efficiency.

As seen in Figure 5A, the reaction between 100 µL of *S. aureus* at 1.0 × 10^5^ CFU/mL and 100 µL of Van-MBs produced the highest capture rate (98%). Upon increasing Van-MBs to 120 µL and 150 μL, the capture rate stays constant. Therefore, the ideal volume to react with the bacteria should be 100 µL of Van-MBs. As demonstrated in Figure 5B, the highest capture rate (99%) was obtained after 55 min of incubation at 37 °C and 200 r/min with 100 µL of *S. aureus* at a concentration of 1.0 × 10^5^ CFU/mL and 100 µL of Van-MBs. The capture rate stayed unchanged after 65 and 75 min of incubation. Therefore, the ideal duration for the incubation between Van-MBs and bacteria should be 55 min. As demonstrated in Figure 5C, after 55 min at 37 °C and 200 r/min with 100 µL of *S. aureus* at a concentration of 1.0 × 10^5^ CFU/mL and 100 µL of Van-MBs, the magnetic separation time was 5 min, and the capture rate was at its highest (99%). As the duration was increased to 6 and 7 min, it was essentially constant. Therefore, it was determined that the ideal duration for separating Van-MBs from the bacteria was 5 min of magnetic separation.

The filter strip’s yellowest color was observed for the same duration for L-alanine-4-nitroaniline solutions at 10 and 15 mg/mL. Therefore, the ideal concentration of the L-alanine-4-nitroaniline solution is 10 mg/mL. Once the ideal concentration had been established, 20, 40, 60, 80, 100, and 150 μL of the 10 mg/mL L-alanine-4-nitroaniline solution were taken to get the ideal volume. The results demonstrated that for both 100 μL and 150 μL L-alanine-4-nitroaniline solutions at the same time, the filter strip’s color was the yellowest. Thus, the optimum *c*oncentration of L-alanine-4-nitroaniline solutions was 10 mg/mL, and its volume was 100 μL.

### 3.5 Specificity and sensitivity

**FIGURE 6:**
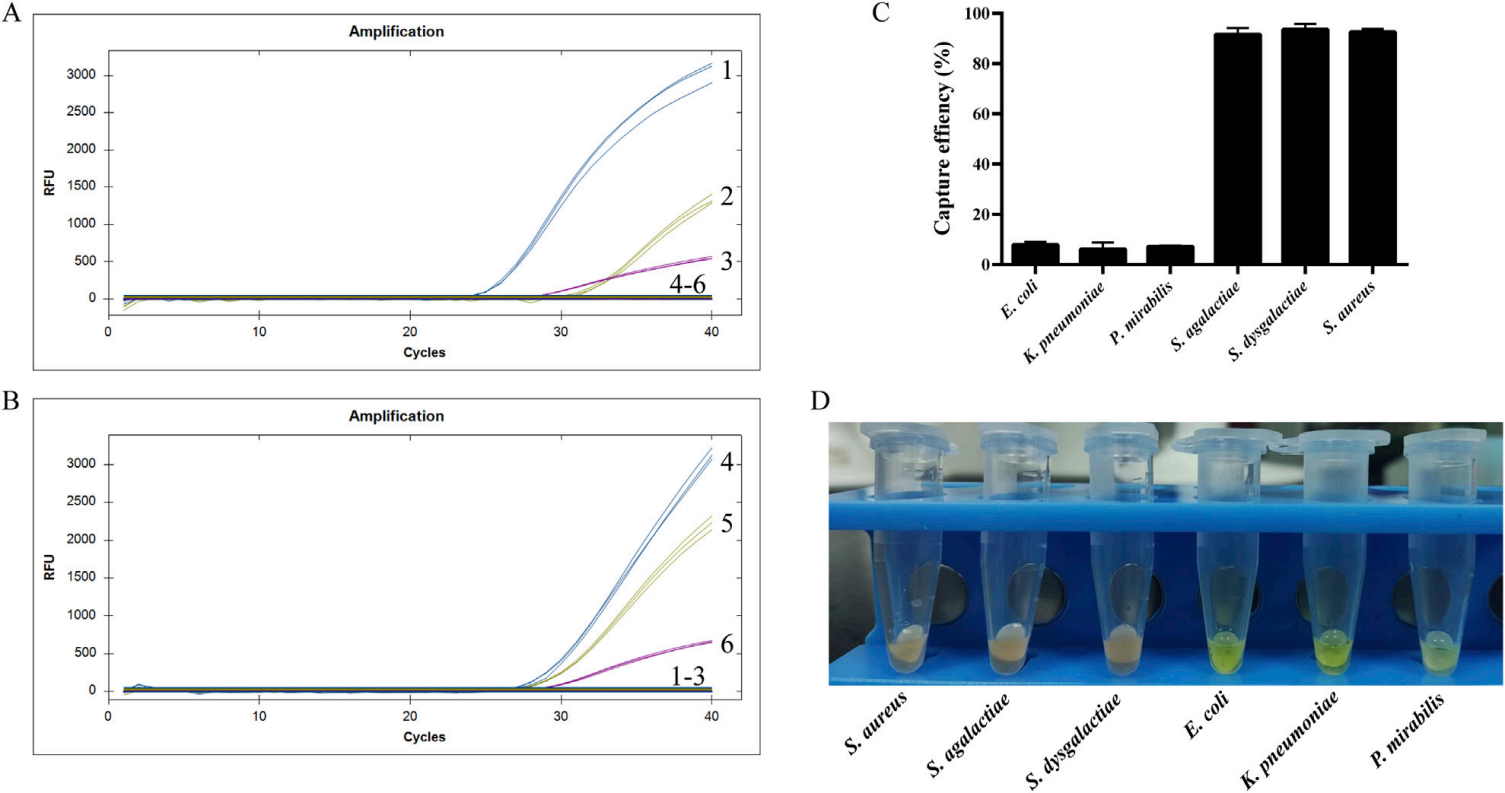
Specificity result. **(A)** qPCR for the resuspended magnetic complexes, **(B)** qPCR for the supernatants, **(C)** Capture efficiency of the resuspended magnetic complexes, **(D)** Aminopeptidase test for the supernatants.

Following the reaction of Van-MBs with *E. coli*, *K. pneumoniae*, *P. mirabilis*, *S. aureus*, *S. agalactiae*, and *S. dysgalactiae*, the supernatant was gathered and magnetic complexes were resuspended. Then the DNA was extracted and analyzed by qPCR. From the qPCR detection results of the resuspended magnetic complexes in Figure 6A *, S. aureus*, *S. agalactiae*, and *S. dysgalactiae* were successfully detected, but not *E. coli*, *K. pneumoniae*, and *P. mirabilis*. The results for the supernatants were the opposite (Figure 6B). Briefly, amplification curves were detected for *E. coli*, *K. pneumoniae*, and *P. mirabilis*, but not for *S. aureus*, *S. agalactiae*, and *S. dysgalactiae.* Furthermore, the resuspended magnetic complexes were spread on the MH for 37 °C and cultured overnight, and the capture rate was calculated based on the number of colonies on the plate, as indicated in Figure 6C. The capture rates of *E. coli*, *K. pneumoniae*, *P. mirabilis*, *S. agalactiae*, *S. dysgalactiae*, and *S. aureus* were 2.8%, 2.5%, 3.0%, 98%, 99%, and 99%, respectively. From the results of the aminopeptidase test strips in the supernatant, *E. coli*, *K. pneumoniae*, and *P. mirabilis* all caused the aminopeptidase test strips to respond by turning them yellow (Figure 6D). However, the aminopeptidase test strips did not turn yellow when they came into contact with *S. agalactiae*, *S. dysgalactiae*, and *S. aureus*. These results indicated that the method established in this study specifically interacts with Gram-negative bacteria.

**FIGURE 7:**
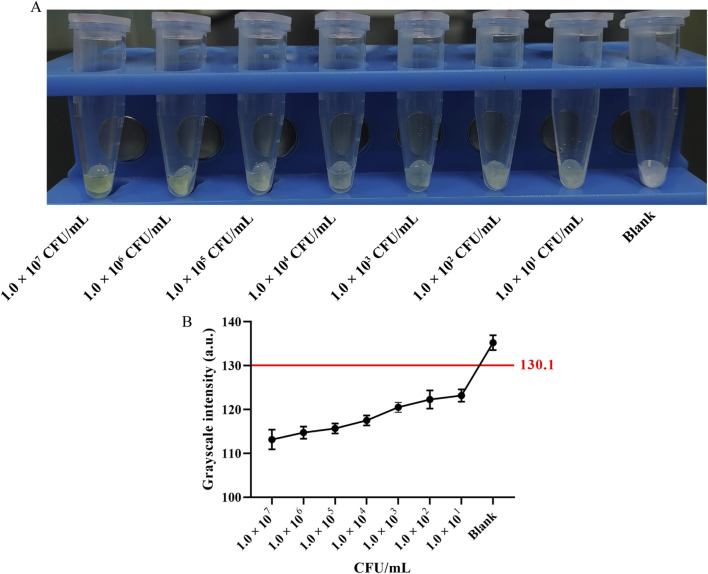
Sensitivity assessment and semi-quantitative analysis. **(A)** Representative photograph of the test strips: concentration of *E. coli* at 1.0 × 10^7^ to 1.0 × 10^1^ CFU/mL, and Blank control. **(B)** Grayscale intensity analysis of the blue channel. Data points represent the mean ± SD of three independent experiments. The red line indicates the threshold (130.1 a.u.) calculated using 3-sigma criterion. The Limit of Detection (LOD) is determined to be 1.0 × 10^1^ CFU/mL.

To assess the sensitivity of the method, 100 μL of Van-MBs and 100 μL of *E. coli* at a concentration of 1.0 × 10^7^ to 1.0 × 10^1^ CFU/mL for 55 min at 37 °C with shaking at 200 r/min, followed by magnetic separation for 5 min. The supernatant was incubated with 100 μL of 10 mg/mL aminopeptidase test strips for 30 min at 37 °C. As seen in Figure 7A, the test strips turned yellow across the entire concentration range tested (1.0 × 10^7^ to 1.0 × 10^1^ CFU/mL). Consistent with the visual observation, the grayscale intensity of the blue channel decreased with increasing bacterial concentration (Figure 7B). The LOD was established using the 3-sigma criterion. As shown in Table 2, the threshold was calculated to be 130.1 a.u. The mean gray value for the 1.0 × 10^1^ CFU/mL sample (123.2 ± 1.4 a.u.) was below the threshold of 130.1 a.u. Therefore, the LOD was determined to be 1.0 × 10^1^ CFU/mL, conclusively demonstrating the high sensitivity of the method.

**TABLE 2:** Semi-quantitative grayscale analysis of the sensitivity assay (n = 3).

Concentration (CFU/mL)	Mean gray value ± SD
Blank	135.2 ± 1.7
3-sigma threshold	130.1
1.0 × 10^1^	123.2 ± 1.4
1.0 × 10^2^	122.3 ± 2.1

The threshold was determined using the 3-sigma criterion: Mean_gray_Blank - 3 × SD_gray_Blank. The LOD, is defined as the lowest concentration with a mean gray value less than this threshold, which corresponds to 1.0 × 10^1^ CFU/mL.

### 3.6 Spiked recovery experiment

To evaluate the clinical applicability of the proposed method, recovery experiments were conducted using three different sample matrices: PBS, saline, and milk. Mixed bacterial standard solutions of varying concentrations were added and tested using this method. As shown in Table 3, the recovery rates for positive bacteria ranged between 75% and 120%, with relative standard deviations (RSD) consistently below 6.4%. In contrast, negative bacteria caused the aminopeptidase test strips to turn yellow. These results indicate that the method has a high potential for separating Gram-positive and Gram-negative bacteria across various matrices.

**TABLE 3:** Recovery of mixed bacteria spiked into different into liquid matrices (n = 3).

Sample	Added(CFU/mL)	Found(CFU/mL)	RSD(%)	Recovery(%)
PBS	1.0 × 10^6^	1.1 × 10^6^	5.8	110
	1.0 × 10^5^	9.1 × 10^4^	6.4	91
	1.0 × 10^4^	9.4 × 10^3^	2.7	94
Saline	1.0 × 10^6^	8.6 × 10^5^	2.1	86
	1.0 × 10^5^	9.9 × 10^4^	5.2	99
	1.0 × 10^4^	9.9 × 10^3^	5.0	99
Milk	1.0 × 10^6^	1.2 × 10^6^	6.3	120
	1.0 × 10^5^	7.5 × 10^4^	5.2	75
	1.0 × 10^4^	8.0 × 10^3^	6.1	80

### 3.7 Clinical sample detection

**FIGURE 8:**
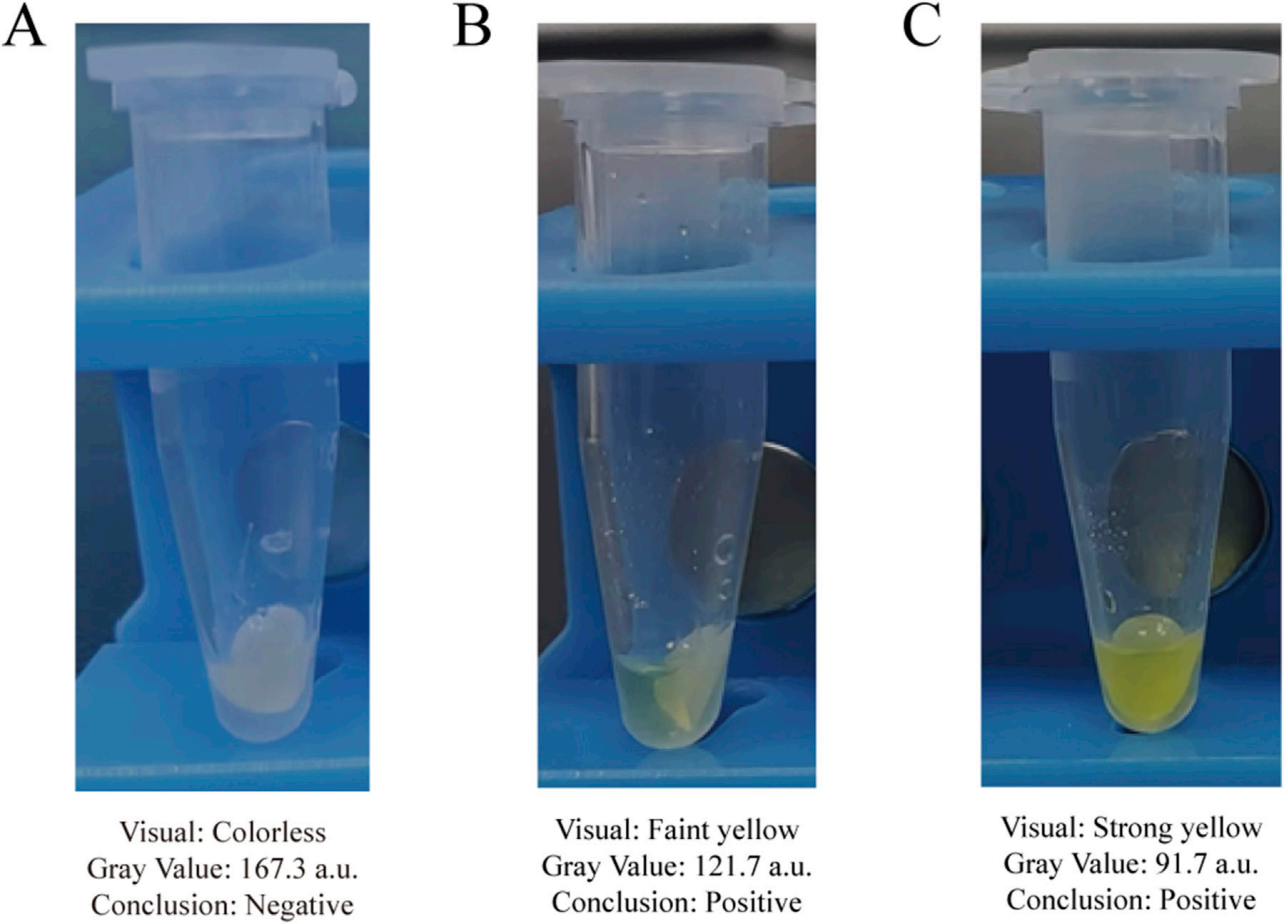
Assessment of representative clinical milk samples using the established grayscale criterion. **(A)** A clinical negative sample with no color change. **(B)** A clinical positive sample with a faint yellow color. **(C)** A clinical positive sample with a distinct yellow color.

When applied to 63 clinical bovine mastitis milk samples, the proposed method detected Gram-negative bacteria in 34 samples, as evidenced by a positive (yellow) colorimetric signal on the aminopeptidase test strips. To provide an objective validation of these visual readouts, we performed a semi-quantitative grayscale analysis on a representative subset of samples. A sample was considered positive for Gram-negative bacteria if the mean gray value of its test strips, measured using the aforementioned ImageJ analysis protocol, was below the statistically defined threshold of 130.1 a.u. (Figure 8). Furthermore, with simple operations and visible outcomes, the entire testing procedure can be finished within 100 min. To validate the clinical accuracy of our method, all 63 samples were analyzed in parallel using a 16-Plex Bovine Mastitis Pathogen Nucleic Acid Detection Kit. Specifically focusing on the Gram-negative bacteria targets in this kit (*Escherichia coli*, *Serratia marcescens*, and *Klebsiella* spp.), the results demonstrated 100% concordance with our method (Table 4). Both methods identified the same 34 samples as positive for Gram-negative bacteria (Supplementary Table S1). This indicates that the Gram-negative bacteria detection method established in this study is not only rapid and visual but also objective and reliable, making it highly suitable for clinical application.

**TABLE 4:** Comparison of Gram-negative bacteria detection in milk samples (n = 63).

Method	Positive samples detected
Van-MBs + Aminopeptidase test	34
Commercial qPCR kit	34

## 4 Discussion

Compared to Gram-positive bacteria, Gram-negative bacteria exhibit inherent resistance to many commonly used antibiotics, such as penicillin, due to their complex outer membrane structure (Arzanlou et al., 2017). More importantly, they can acquire resistance through various mechanisms, including plasmid-mediated gene transfer, regulation of outer membrane protein expression, and enhanced activity of efflux pumps. The resulting resistance phenotypes are highly complex and variable, contributing to the high prevalence of multidrug-resistant (MDR) Gram-negative bacteria (Domalaon et al., 2018; Macesic et al., 2025). According to data released in 2024 by the China Antimicrobial Surveillance Network (CHINET), the overall ratio of Gram-positive to Gram-negative bacteria clinically isolated from various hospitals across different regions of China is approximately 3:7. Key Gram-negative bacteria such as *E*. *coli*, *K*. *pneumoniae*, *P*. *aeruginosa*, and *A*. *baumannii* rank among the top five and demonstrate significant resistance to multiple commonly used antibiotics, which further complicates clinical treatment. Furthermore, many common foodborne pathogens, including *E*. *coli*, *K*. *pneumoniae*, and *Salmonella*, are Gram-negative bacteria. Therefore, the development of rapid detection technologies suitable for the on-site identification of Gram-negative bacteria has become an urgent necessity in the current field of microbiology.

In recent years, there has been an increasing focus on covalently binding recognition elements to magnetic beads to enhance the capture of target bacteria (Deng et al., 2021; Li et al., 2022; Yu et al., 2022; Du et al., 2024). A prominent example of this strategy is the use of vancomycin, which is known for its ability to interact with D-Ala-D-Ala peptide moieties in the cell walls of Gram-positive bacteria through five hydrogen bonds, and has been extensively utilized as a recognition element for capturing these bacteria. Numerous studies have employed vancomycin-functionalized magnetic beads to isolate target bacteria from complex matrices, such as blood, urine, and water samples (Kell et al., 2008). The applications of this platform are diverse. For instance, utilizing vancomycin-functionalized magnetic beads and quantifying the capture of bacteria through the biochemical luminescence signal of intracellular adenosine triphosphate, this method demonstrates a linear detection range of 1.0 × 10^2^ to 1.0 × 10^7^ CFU/mL for four types of Gram-positive bacteria, with a detection limit of 33 CFU/mL (Su et al., 2017). Another approach combined vancomycin-functionalized magnetic beads with FITC-labeled IgG and flow cytometry to detect *S. aureus*, within 120 min with an LOD of 3.3 × 10^1^ CFU/mL (Meng et al., 2017b). Additionally, using vancomycin-modified nanomaterials with MALDI-MS yielded the minimum detection limit for *S. saprophyticus* and *S. aureus* in urine, which was determined to be 7 × 10^4^ CFU/mL (Lin et al., 2005). Furthermore, studies have covalently linked vancomycin to FePt magnetic nanoparticles, enabling the detection of vancomycin-resistant *Enterococci* and other Gram-positive bacteria through multivalent ligand-receptor interactions, with a detection limit of 1 CFU/mL (Gu et al., 2003). Vancomycin-functionalized magnetic beads, in conjunction with alkaline phosphatase-tagged rabbit immunoglobulin, were utilized to enrich *S. aureus* (Yang et al., 2016), while vancomycin-functionalized PEGylated magnetic nanoparticles were employed to recognize and capture *L. monocytogenes* (Meng et al., 2017a). Consistent with these reported successes, the vancomycin-functionalized magnetic beads synthesized in our study effectively separated and enriched Gram-positive bacteria from raw milk samples within 60 min, thereby achieving effective separation of Gram-positive and Gram-negative bacteria.

Aminopeptidase detection technology is based on the differences in bacterial enzyme activity to enable rapid bacteria screening. For instance, one study used a combination of 3-amino-N-(3-fluorophenyl) propanamide and the substrate β-Alanine Aminopeptidase to detect *P*. *aeruginosa* in sputum samples from patients with cystic fibrosis (Thompson et al., 2020). β-alanine aminopeptidase is exclusively present in certain specific Gram-negative bacteria that can utilize β-alanine, such as *P*. *aeruginosa*, *B*. *cepacia*, and *S*. *marcescens* (Váradi et al., 2019). In contrast, L-alanine aminopeptidase is found in nearly all Gram-negative bacteria. The specificity of this enzyme distribution provides a robust basis for differentiating Gram-negative bacteria from Gram-positive bacteria. Currently detection of L-alanine aminopeptidase primarily relies on specific substrates, with L-alanine-4-nitroaniline being the most common (Cellier et al., 2014). When Gram-negative bacteria are present, L-alanine aminopeptidase decomposes L-alanine-4-nitroaniline into 4-nitroaniline and L-alanine, resulting in a yellow color change due to the formation of 4-nitroaniline. L-alanine-4-nitroaniline can be coated onto a test strip, allowing for rapid detection of Gram-negative bacteria through this color change. Compared to traditional Gram staining, the L-alanine aminopeptidase-based detection technique significantly simplifies the workflow. Despite its advantages, the technique faces the significant challenge of sample matrix interference. In our study, the use of vancomycin-functionalized magnetic beads for sample pretreatment enabled the selective retention of only the supernatant containing Gram-negative bacteria from complex samples, such as milk, for test strip detection. This approach minimized the chance of non-target microorganisms interacting with L-alanine aminopeptidase, thereby reducing the false-positive rate and enhancing the repeatability of the results. Furthermore, it increased the relative concentration of Gram-negative bacteria, which effectively lowered the detection limit of the assay.

The method’s accuracy was confirmed by its perfect agreement with a commercial multiplex qPCR kit, and its objectivity was ensured by a semi-quantitative analysis that defined the detection limit via the 3-sigma criterion. The absence of any discrepancies strongly indicates the high specificity and reliability of our method in a clinical setting. With a total time of 100 min and a visual readout, this technique establishes itself as an effective screening tool that requires no complex instruments or specialized training to obtain the result.

## 5 Conclusion

In summary, we developed a sensitive and rapid method for detecting Gram-negative bacteria by combining Van-MBs and aminopeptidase test strips. This assay achieves a detection limit of 10^1^ CFU/mL and can be completed within 100 min. It is a rapid, simple, cost-effective, and visible platform for identifying Gram-negative bacteria in clinical samples. We anticipate that this method will provide crucial guidance for the rapid and accurate selection of antibiotics to treat bovine mastitis. Furthermore, the method holds potential for identifying bacterial pathogen types in other disease samples.

## Data Availability

All data generated or analyzed during this study are included in this published article and its Supplementary Information files, further inquiries can be directed to the corresponding author.
