# Supplementary material for: Separation and detection of Gram-negative bacteria via vancomycin-functionalized magnetic beads and aminopeptidase test strips
Source: Front Bioeng Biotechnol. 2025 Nov 20;13:1712799. doi: 10.3389/fbioe.2025.1712799 (PMC12676118; doi:10.3389/fbioe.2025.1712799)
Supplement: Supplementary file 1 [file Table1.docx]

Supplementary Material

**Supplementary Table 1 Detailed comparison of detection results for Gram-negative bacteria.**

| Sample | Van-MBs+  Aminopeptidase Test | *Escherichia coli* | *Serratia marcescens* | *Klebsiella spp.* | Overall Commercial qPCR Kit Result |
| --- | --- | --- | --- | --- | --- |
| 180314 | Positive | + | - | + | Positive |
| 170908 | Positive | + | + | - | Positive |
| 190104 | Negative | - | - | - | Negative |
| 190085 | Positive | + | - | - | Positive |
| 211187 | Positive | + | - | - | Positive |
| 200822 | Negative | - | - | - | Negative |
| 190338 | Positive | + | - | + | Positive |
| 180809 | Positive | + | - | - | Positive |
| 180344 | Negative | - | - | - | Negative |
| 190330 | Positive | + | - | - | Positive |
| 180314 | Positive | + | - | - | Positive |
| 201309 | Positive | + | - | - | Positive |
| 181193 | Positive | + | - | - | Positive |
| 211314 | Positive | + | - | - | Positive |
| 211248 | Positive | + | + | + | Positive |
| 211152 | Positive | + | - | - | Positive |
| 19010 | Negative | - | - | - | Negative |
| 14325 | Positive | + | - | - | Positive |
| 181226 | Positive | + | - | - | Positive |
| 190704 | Negative | - | - | - | Negative |
| 200825 | Positive | + | - | - | Positive |
| 201027 | Positive | + | - | - | Positive |
| 190001 | Negative | - | - | - | Negative |
| 200409 | Positive | + | - | - | Positive |
| 190151 | Negative | - | - | - | Negative |
| 210923 | Positive | + | + | + | Positive |
| 191032 | Positive | + | - | - | Positive |
| 190742 | Negative | - | - | - | Negative |
| 181239 | Positive | + | - | - | Positive |
| 160408 | Negative | - | - | - | Negative |
| 180923 | Positive | + | - | - | Positive |
| 191455 | Negative | - | - | - | Negative |
| 190436 | Negative | - | - | - | Negative |
| 200908 | Negative | - | - | - | Negative |
| 190580 | Negative | - | - | - | Negative |
| 210562 | Negative | - | - | - | Negative |
| 180427 | Negative | - | - | - | Negative |
| 180547 | Positive | + | - | - | Positive |
| 191088 | Negative | - | - | - | Negative |
| 211234 | Positive | - | - | + | Positive |
| 211301 | Negative | - | - | - | Negative |
| 18030 | Negative | - | - | - | Negative |
| 200909 | Positive | + | - | - | Positive |
| 180335 | Negative | - | - | - | Negative |
| 171114 | Positive | + | - | + | Positive |
| 200163 | Positive | + | - | + | Positive |
| 191221 | Negative | - | - | - | Negative |
| 190547 | Positive | + | + | + | Positive |
| 181114 | Positive | + | - | + | Positive |
| 210759 | Negative | - | - | - | Negative |
| 170304 | Negative | - | - | - | Negative |
| 181153 | Negative | - | - | - | Negative |
| 211313 | Positive | + | - | + | Positive |
| 210923 | Positive | - | + | + | Positive |
| 180224 | Negative | - | - | - | Negative |
| 190789 | Negative | - | - | - | Negative |
| 200942 | Positive | + | - | + | Positive |
| 190580 | Positive | + | - | + | Positive |
| 200812 | Positive | - | - | + | Positive |
| 180998 | Negative | - | - | - | Negative |
| 210866 | Negative | - | - | - | Negative |
| 190443 | Negative | - | - | - | Negative |
| 211182 | Negative | - | - | - | Negative |
| Total | 34 | 31 | 5 | 14 | 34 |

**Note: “+”** denotes a positive result; **“-”** denotes a negative result.

The Van-MBs+Aminopeptidase Test Method provides a composite result for the presence of any Gram-negative bacteria.

The Overall Commercial qPCR Kit Result is positive if any of the three individual Gram-negative targets is positive.
